# Supplementary material for: Horses in the Early Medieval (10th–13th c.) Religious Rituals of Slavs in Polish Areas—An Archaeozoological, Archaeological and Historical Overview
Source: Animals (Basel). 2022 Sep 3;12(17):2282. doi: 10.3390/ani12172282 (PMC9454541; doi:10.3390/ani12172282)
Supplement: Supplementary file 1 [file animals-12-02282-s001.zip › animals-1879378-supplementary.pdf]

Table S1
List of sites with horse remains analyzed in the project entitled 'Horse in Poland in the Early Piasts and Internal Fragmentation'

No	Locality/site	Chronology	Province	Community
1.	Baldram, 1	10th – 11th	pomorskie	Kwidzyn
2.	Bardy	7th – 9th/10th	zachodniopomorskie	Dygowo
3.	Biała Góra, 3	late 12th – mid 15th	pomorskie	Sztum
4.	Białogard, 1	late 6th – mid 13th	zachodniopomorskie	loco
5.	Bielsk Podlaski, Zamkowa	10th – 13th	podlaskie	loco
6.	Biskupin, 4	10th – 13th	wilekopolskie	Gąsawa
7.	Bnin	10th – 15th	wielkopolskie	Kórnik
8.	Bobrowo, 1	9th – mid 11th	kujawsko – pomorskie	Bobrowo
9.	Bocheń	8th – 9th	łódzkie	Łowicz
10.	Bonikowo, 1	8th – 10th	wielkopolskie	Kościan
11.	Bonikowo, 2	6th – 10th	wielkopolskie	Kościan
12.	Brodnica – Michałowo, 1	10th – 13th	kujawsko – pomorskie	Brodnica
13.	Brodno	10th – 12th	dolnośląskie	Środa Śląska
14.	Bródno Stare, 1	10th – 13th	mazowieckie	Warszawa
15.	Bruszczewo, 12	9th – mid 10th	wielkopolskie	Śmigiel
16.	Bruszczewo, 13	mid 11th – mid 10th	wielkopolskie	Śmigiel
17.	Brzeźno, 1	7th – 10th/11th	zachodniopomorskie	Barwice
18.	Busówno	9th – 13th	lubelskie	Wierzbica
19.	Bydgoszcz, stan. 1	11th – 14th	kujawsko – pomorskie	loco
20.	Cedynia, 1	9th – 12th	zachodniopomorskie	loco
21.	Charzykowy, 1 – 2	8th – 10th	pomorskie	Chojnice
22.	Chełm, Wysoka Górka, S	13th – 14th	lubelskie	loco
23.	Chełmno, 4 (434)	9th – late 13th	wielkopolskie	Dąbie
24.	Chlebnia, 3	11th – 12th	mazowieckie	Grodzisk Mazowiecki
25.	Chmielno, 1	10th – 13th	pomorskie	Chmielno
26.	Chojnice, 1	8th – 10th	pomorskie	loco
27.	Chycina, 19	10th – 13th	lubuskie	Bledzew
28.	Ciepłe, IV	11th	pomorskie	Gniew

No	Locality/site	Chronology	Province	Community
29.	Czeladź Wielka	6th – 13th	dolnośląskie	Wąsosz
30.	Czerchów, 1	9th – 11th	łódzkie	Ozorków
31.	Czermno Kolonia, 3	12th – 13th	lubelskie	Tyszowce
32.	Czermno, 1 – 3	late 10th – 13th	lubelskie	Tyszowce
33.	Czersk, 1	9th/10th – 14th	mazowieckie	loco
34.	Czerwona Wieś, 1	late 10th – mid 11th	wielkopolskie	Krzywiń
35.	Czułczyce, 2	9th – 14th	lubelskie	Sawin
36.	Daleszyn, 2	9th/10th – 13th	wielkopolskie	Gostyń
37.	Dąbrówka, 1	13th – 14th	wielkopolskie	Dopiewo
38.	Dąbrówka, 2	k. 8th – mid 11th	wielkopolskie	Dopiewo
39.	Derczewo, 3	late 6th – mid 8th	zachodniopomorskie	Myślibórz
40.	Dobromierz	9th – 10th	dolnośląskie	loco
41.	Dobrzyń nad Wisłą, 1	9th – 15th	kujawsko – pomorskie	loco
42.	Dziekanowice, 22	late 10th – 12th	wielkopolskie	Łubowo
43.	Elbląg – Stare Miasto	late 13th	warmińsko – mazurskie	Elblag
44.	Elbląg – Zamek, 1	end 13th – 19th	warmińsko – mazurskie	Elbląg
45.	Gardziec	9th – 12th	zachodniopomorskie	Przelewice
46.	Gdańsk, 1 (1000 – 1020)	11th – 13th	pomorskie	loco
47.	Gdańsk, 10 (1230 – 1308)	9th – 13th	pomorskie	loco
48.	Gdańsk, 2 (1210 – 1230)	13th – 14th	pomorskie	loco
49.	Gdańsk, 4 (1255 – 1275)	13th	pomorskie	loco
50.	Gdańsk, Plac Heweliusza	11th – 13th	pomorskie	loco
51.	Gdańsk, ul. Grodzka	late 11th – mid14th	pomorskie	loco
52.	Gdańsk, ul. Olejarna	13th – 15th	pomorskie	loco
53.	Giecz	9th – 14th	wielkopolskie	Dominowo
54.	Gilów	9th – 10th	dolnośląskie	Niemcza
55.	Głuchowo, 1	9th – 1 half 13th	wielkopolskie	Komorniki
56.	Gniew – Stare Miasto, 2	late 11th – 13th	pomorskie	Gniew
57.	Gniew, 3	late 11th – 13th	pomorskie	Gniew

No	Locality/site	Chronology	Province	Community
58.	Gniew, 5	end 9th – 10th/11th	pomorskie	Gniew
59.	Gniezno, 13b	late 10th – mid 11th	wielkopolskie	Gniezno
60.	Gniezno, 14	10th – 13th	wielkopolskie	Gniezno
61.	Gniezno, 15	late 9th – 13th	wielkopolskie	Gniezno
62.	Gniezno, 15a – d	9th – 13th	wielkopolskie	Gniezno
63.	Gniezno, 17a	11th/12th – 12th	wielkopolskie	Gniezno
64.	Gniezno, 22	early 10th – 13th/14th	wielkopolskie	Gniezno
65.	Gniezno, 43	10th – 13th	wielkopolskie	loco
66.	Gniezno, 5	10th – 13th	wielkopolskie	Gniezno
67.	Gniezno, 51	11th – 12th	wielkopolskie	loco
68.	Gniezno, 7a	11th – 12th	wielkopolskie	Gniezno
69.	Gniezno, 7c	9th – 13th/14th	wielkopolskie	loco
70.	Gniezno – Piotrowo, 40	early 10th – 14th	wielkopolskie	loco
71.	Golęczewo, 26	late 11th – mid 12th	wielkopolskie	Suchy Las
72.	Gorzędziej, 1	9th – 13th/14th	pomorskie	Subkowy
73.	Góra, 1	6th/7th – 8th/9th	wielkopolskie	Pobiedziska
74.	Góra, 2	6th/7th – 8th/9th	wielkopolskie	Pobiedziska
75.	Górzyca, 1	7th – late 12th	lubuskie	loco
76.	Górzyca, 20	10th – early 11th	lubuskie	loco
77.	Grążawy, 1	11th/12th – half 13th	kujawsko – pomorskie	Bartniczka
78.	Grążawy, 2	11th/12th – half 13th	kujawsko – pomorskie	Bartniczka
79.	Grodzisko, 1	8th? – 13th?	warmińsko – mazurskie	Banie Mazurskie
80.	Grodziszcze, 1	9th – half 10th	lubuskie	Świebodzin
81.	Gronowo, 1	8th – 11th/12th	kujawsko – pomorskie	Lubicz
82.	Gronowo, 2	late 7th – 11th/12th	kujawsko – pomorskie	Lubicz
83.	Grotniki, 6	11th – 13th	wielkopolskie	Włoszakowice
84.	Grudusk, 1	10th – 14th	mazowieckie	loco
85.	Grudziądz, 1	11th	kujawsko – pomorskie	loco
86.	Grzybowo, 1	mid 11th – 11th/12th	wielkopolskie	Września

No	Locality/site	Chronology	Province	Community
87.	Gutowo, 1	12th/13th	warmińsko – mazurskie	Lubawa
88.	Gwieździn, 1	9th – 12th w	pomorskie	Rzeczenica
89.	Horodyszcze, 3	8th – 11th	lubelskie	Wisznice
90.	Iłża, 1	11th – 12th	mazowieckie	loco
91.	Iłża, 2	11th – 12th	mazowieckie	loco
92.	Inowrocław, 18	13th – 14th	kujawsko – pomorskie	loco
93.	Inowrocław, 19	13th – 14th	kujawsko – pomorskie	loco
94.	Inowrocław, 20	11th – 12th	kujawsko – pomorskie	loco
95.	Inowrocław, 2a	11th – 12th	kujawsko – pomorskie	loco
96.	Janów Pomorski, 1	late 8th – mid 10th	warmińsko – mazurskie	Elbląg
97.	Jastrowo – Ostrolesie, 1	9th – mid 10th	wielkopolskie	Szamotuły
98.	Jedwabno, 1	11th – 14th	kujawsko – pomorskie	Lubicz
99.	Jedwabno, 2	7th/8th – 11th/12th	kujawsko – pomorskie	Lubicz
100.	Jeziorko	10th – mid13th	warmińsko – mazurskie	Ryn
101.	Jordanowo, 7	11th – early 13th	lubuskie	Świebodzin
102.	Junkrowy, 1	8th – mid13th	pomorskie	Skarszewy
103.	Kaczyce, 23	11th – 13th	świętokrzyskie	Lipnik
104.	Kalisz – Zawodzie	9th – 14th	wielkopolskie	loco
105.	Kałdus, 1	late 10th – mid 13th	kujawsko – pomorskie	Chełmno
106.	Kałdus, 2	late 7th – mid 13th	Kujawsko – pomorskie	Chełmno
107.	Kałdus, 3	late 7th – 13th/14th	kujawsko – pomorskie	Chełmno
108.	Kałdus, 4	10th/11th – mid 11th	kujawsko – pomorskie	Chełmno
109.	Kamień Pomorski	10th – 13th	zachodniopomorskie	loco
110.	Kaszowo, 1	late 10th – 11th	dolnośląskie	Milicz
111.	Kąsinowo	11th	wielkopolskie	Szamotuły
112.	Kędrzyno 1	late 8th – 9th	zachodniopomorskie	Siemyśl
113.	Klenica, 4	9th – 11th	lubuskie	Bojadła
114.	Kołobrzeg – Budzistowo 1	half 9th – early 11th	zachodniopomorskie	loco
115.	Konikowo, 1 i 2	early medieval	warmińsko – mazurskie	Gołdap

No	Locality/site	Chronology	Province	Community
116.	Kozanki Podleśne 1	9th – 12th	łódzkie	Świnice Warckie
117.	Kraków – Sukiennice	11th/12th	małopolskie	loco
118.	Krępsk	8th – 10th	pomorskie	Człuchów
119.	Krosno Odrzańskie	11th – 13th	lubuskie	loco
120.	Kruszwica, 2	9th – 14th	wielkopolskie	loco
121.	Kruszwica, 4	10th – 13th	wielkopolskie	loco
122.	Kruszwica 5	9th – 13th	wielkopolskie	loco
123.	Kruszwica, 9	8th – 12th	kujawsko – pomorskie	loco
124.	Krzyżowniki, 16	late 10th – early 12th	wielkopolskie	loco
125.	Kulczyn Kolonia, 8	10th – 11th	lubelskie	Hańsk
126.	Ląd, 1	10th – 13th	wielkopolskie	Lądek
127.	Legnica	7th/8th	dolnośląskie	loco
128.	Lembarg, 6	late10th – mid 11th	kujawsko – pomorskie	Jabłonowo Pomorskie
129.	Lubieszewo, 1	10th	zachodniopomorskie	Złocieniec
130.	Lubin 1	11th – 14th	zachodniopomorskie	loco
131.	Lubiń, 1	late 11th – 14th	wielkopolskie	Krzywiń
132.	Lubiszewo	9th – 11th	pomorskie	Tczew
133.	Lubniewice, 10	late 8th – 11th	lubuskie	Lubniewice
134.	Lubniewice, 9	late 8th – mid 12	lubuskie	Lubniewice
135.	Lubrza, stan 42	9th – mid 10th	lubuskie	Lubrza
136.	Łącko, 5	7th – mid 10th	kujawsko – pomorskie	Pakość
137.	Łekno, 3	9th – 14th	wielkopolskie	Wągrowiec
138.	Łubowo, 57	7th/I10th – 11th/12th	wielkopolskie	loco
139.	Malbork	10t10/11th – 13th	pomorskie	loco
140.	Mietlica, 2/3	7th – 11th	kujawsko – pomorskie	Kruszwica
141.	Mietlica, 3	7th – 11th	kujawsko – pomorskie	Kruszwica
142.	Mietlica, 4	7th – 11th	kujawsko – pomorskie	Kruszwica
143.	Mietlica, 9	7th – 11th	kujawsko – pomorskie	Kruszwica
144.	Międzyrzecz 1 – Zamek	late8th – 13th	lubuskie	loco

No	Locality/site	Chronology	Province	Community
145.	Milicz	late 10th – 13th	dolnośląskie	loco
146.	Moraczewo, 1	mid 10th – 11th	wielkopolskie	Łubowo
147.	Mrówki	13th/14th	wielkopolskie	Wilczyn
148.	Myślibórz, 1	8th/9th – late13th	zachodniopomorskie	loco
149.	Nakło nad Notecią	8th – 12th	kujawsko – pomorskie	loco
150.	Napole, 1	late 10th – 1 half 14th	kujawsko – pomorskie	Kowalewo Pomorskie
151.	Napole, 1B	late 7th – early 13th	kujawsko – pomorskie	Kowalewo Pomorskie
152.	Napole, 6	late 7th – early 13th	kujawsko – pomorskie	Kowalewo Pomorskie
153.	Naszacowice, 1	8th – 11th	małopolskie	Podegrodzie
154.	Nętno, 1	10th – mid 11th	zachodniopomorskie	Drawsko Pomorskie
155.	Nętno, 1b	10th – mid 11th	zachodniopomorskie	Drawsko Pomorskie
156.	Niemcza	8th – 10th	dolnośląskie	loco
157.	Nowa Wieś, 12	6th – 8th	lubuskie	Bledzew
158.	Nowiniec, 2	9th – 10th	lubuskie	Lubsko
159.	Nowy Dworek, 10	late 9th – early 10th	lubuskie	Świebodzin
160.	Nowy Dworek, 27	mid 10th – mid 12th	lubuskie	Świebodzin
161.	Nowy Dworek, 7	7th/8th – 9th	lubuskie	Świebodzin
162.	Objezierze, 1	9th – mid 11th	wielkopolskie	Oborniki
163.	Obrowo, 1	late 7th – mid 10th	wschdniopomorskie	Kęsowo
164.	Olsztyn Las Miejski, CIV	8th – 9th	warmińsko – mazurskie	loco
165.	Opole – Ostrówek, 1	12th – 13th	opolskie	loco
166.	Osiek Rypiński, 1	12th	kujawsko – pomorskie	Osiek
167.	Ostrów Lednicki – Rybitwy, 3a	10th – 14th	wielkopolskie	Łubowo
168.	Ostrów Lednicki, 1	9th – 14th	wielkopolskie	Łubowo
169.	Ostrów Lednicki, 2	9th – 13th/14th	wielkopolskie	Łubowo
170.	Otanów 27	10th – 11th	zachodniopomorskie	Myślibórz
171.	Otomin, 1	9th – mid 10th	pomorskie	Kolbudy
172.	Owidz, 1	9th – 12th	pomorskie	Starogard Gdański
173.	Parsęcko, 2	late 11th	zachodniopomorskie	Szczecinek

No	Locality/site	Chronology	Province	Community
174.	Pelplin – Maciejewo	8th – 9th	pomorskie	Pelplin
175.	Pęczniew, 3	11th – 12th	łódzkie	loco
176.	Pień, 2	late 10th – early 11th	kujawsko – pomorskie	Dąbrowa Chełmińska
177.	Pień, 9	10th/11th	kujawsko – pomorskie	Dąbrowa Chełmińska
178.	Płochocin, 6	late 11 – mid 12th	Kujawsko – pomorskie	Warlubie
179.	Płochocinek, 14A	11th – 12th	Kujawsko – pomorskie	Warlubie
180.	Płochocinek, 14B	late 11th – late 12th	Kujawsko – pomorskie	Warlubie
181.	Płock	9th – 13th	mazowieckie	loco
182.	Płońsk 7	11th – 13th	mazowieckie	loco
183.	Podgórze, 1	eraly medieval	lubelskie	Chełm
184.	Podzamcze, 1	9th – 13th	pomorskie	Kwidzyn
185.	Poganowo, 4	11th – 12th	warmińsko – mazurskie	Kętrzyn
186.	Połupin, 2	8th – 9th	lubuskie	Dąbie
187.	Poniec, 1	late 10th – mid 11th	wielkopolskie	loco
188.	Powodów II, 5	mid10th – 12th	łódzkie	Wartkowice
189.	Poznań – Ostrów Tumski (Katedra)	10th – 11th	wielkopolskie	loco
190.	Poznań – Ostrów Tumski (ogród arcybiskupi	late 11th – 12th	wielkopolskie	loco
191.	Poznań – Ostrów Tumski (Plac Katedralny)	late10th – mid 11th	wielkopolskie	k. X – poł. XI w.
192.	Poznań – Ostrów Tumski, 10	mid 12th – 15th	wielkopolskie	loco
193.	Poznań – Ostrów Tumski, 17	half 10th – late 13th	wielkopolskie	loco
194.	Poznań – Ostrów Tumski, NMP	10th – 13th	Wielkopolskie	loco
195.	Poznań – Ostrów Tumski, ul. Posadzego 5	half 10th – 15th	wielkopolskie	loco
196.	Poznań – Stary Rynek 43	13th – 15th	wielkopolskie	loco
197.	Poznań – Stary Rynek 48	13th – 15th	wielkopolskie	loco
198.	Poznań – Stary Rynek S	mid13th – 15th	wielkopolskie	loco
199.	Poznań – Śródka, 26	late 10 – 15th	wielkopolskie	loco
200.	Poznań – Zagórze (Alumnat)	10th/11th – late 11th	wielkopolskie	loco

No	Locality/site	Chronology	Province	Community
201.	Poznań, ul Garbary 75/77	11th/12th – 14th	wielkopolskie	loco
202.	Poznań, ul. Wieżowa 2 – 4	9th – 11th10	wielkopolskie	loco
203.	Poznań, ul. Wodna	14th – 15th	wielkopolskie	loco
204.	Poznań, ul. Wodna 13	mid13th – 14th	wielkopolskie	loco
205.	Poznań, ul. Zagórze	mid10th – late 13th	wielkopolskie	loco
206.	Poznań – Wilda 163	10th – 15th	wielkopolskie	loco
207.	Poznań – Wilda 164	10th – 14th	wielkopolskie	loco
208.	Półtusk, 1	late 13th – 14th	mazowieckie	loco
209.	Praslity, 21	6th – 11th	warmińsko – mazurskie	Nowe Miasto
210.	Pruszcz Gdański	late 10th – 11th	pomorskie	loco
211.	Przełazy, 6	late 9th – 12th	lubuskie	Lubrza
212.	Przemyśl, 83	mid 10th	przemyskie	loco
213.	Przytok, 1	late 8th – 1 half 9th	lubuskie	Zabór
214.	Racot, 18	8th – 12th	wielkopolskie	Kościan
215.	Racot, 25	12th – 12th/13th	wielkopolskie	Kościan
216.	Radacz, 1	late 8th – mid 10th	zachodniopomorskie	Szczecinek
217.	Radacz, 2	late 9th – 80. 10	zachodniopomorskie	Szczecinek
218.	Radłowice, 22	early mediewal	dolnośląskie	Domaniów
219.	Radom, 2	8th – 12th	mazowieckie	loco
220.	Radzim, 1	9th/10th – mid 14th	wielkopolskie	Murowana Goślina
221.	Robity, 3	early medieval	warmińsko – mazurskie	Pasłęk
222.	Rudki	9th – mid 10th	wielkopolskie	Szamotuły
223.	Ruska Wieś, 13	10 – 12th/13th	warmińsko – mazurskie	Mrągowo
224.	Ryczyn	late10th – 11th/12th	dolnośląskie	Oława
225.	Ryczyn Duży	10 – 13th	dolnośląskie	Oława
226.	Ryczyn Mały	9th – 12th	dolnośląskie	Oława
227.	Ryczyn, 1	late 10th – 13th	dolnośląskie	Oława
228.	Rzymówka	8th – 11th	dolnośląskie	Złotoryja
229.	Sandomierz Collegium Gostomianum	early medieval	świętokrzyskie	loco

No	Locality/site	Chronology	Province	Community
230.	Sandomierz, 1	10th – 13th	świętokrzyskie	loco
231.	Sandomierz – Zamek II	late 8th – mid 13th	świętokrzyskie	loco
232.	Santok, 1	late 8th – mid 13th	lubuskie	loco
233.	Sąsiadka	11th	lubelskie	Sułów
234.	Siemowo, 1	7th/8th – 10th	wielkopolskie	Gostyń
235.	Sieradz, 1	11th	łódzkie	loco
236.	Skarszewy	9th – 10th	pomorskie	Skarszewy
237.	Skęczniew	8th – 13th	wielkopolskie	Dobra
238.	Słoszewy, 1	late 14th – mid 15th	kujawsko – pomorskie	Bobrowo
239.	Słupsk, 1	7th – 12th	pomorskie	loco
240.	Słupsk, 3	13th/14th	pomorskie	loco
241.	Sopot, 1	8th – mid 10th	pomorskie	loco
242.	Spławie, 1	late 10th	wielkopolskie	Kołaczkowo
243.	Spławie, 2	9th – mid 10th	wielkopolskie	Kołaczkowo
244.	Stare Drawsko (gród)	9th – 15th	zachodniopomorskie	Czaplinek
245.	Stargard – Osetno	late 8th – mid 9th	zachodniopomorskie	loco
246.	Stargard – podgrodzie	mid 9th – late 13th	zachodniopomorskie	loco
247.	Stargard, 11a	late 13th – early 15th	zachodniopomorskie	loco
248.	Stargard, 11a	10th – 12th	zachodniopomorskie	loco
249.	Starorypin, 1A	late 11th – mid 13th	kujawsko – pomorskie	Rypin
250.	Starorypin, 1B	late 13th – early 14th	kujawsko – pomorskie	Rypin
251.	Starorypin, 3	late 11th – 14th	kujawsko – pomorskie	Rypin
252.	Starorypin, 4	mid 11th – early 12th	kujawsko – pomorskie	Rypin
253.	Stary Zamek, 6	8th – 9th	dolnośląskie	Sobótka
254.	Staświny, 1	10th – 13th	warmińsko – mazurskie	Miłki
255.	Stołpie, 1	13th	lubelskie	Chełm
256.	Strachów	late 10th/11th	dolnośląskie	Sobótka
257.	Stradów, 1	late 9th – mid 11th	świętokrzyskie	Czarnocin
258.	Suchy Las, 12	late 10th – mid 13th	wielkopolskie	loco

No	Locality/site	Chronology	Province	Community
259.	Suchy Las, 6	late 10th – mid 14th	wielkopolskie	loco
260.	Sypniewo	10th/11th	mazowieckie	loco
261.	Sypniewo, 1	early 10th – late 11th	mazowieckie	loco
262.	Szarlej, 5	9th	kujawsko – pomorskie	Kruszwica
263.	Szczecin – Mścięcino	9th – 12th	zachodniopomorskie	loco
264.	Szczecin – Podzamcze	11th/12th – 13th	zachodniopomorskie	loco
265.	Szczecin – Rynek warzywny	10th – mid 13th	zachodniopomorskie	loco
266.	Szczecin – Stare Miasto, VI	9th – 13th	zachodniopomorskie	loco
267.	Szczecin – Zamek, wp IV – V	7th – 13th	zachodniopomorskie	loco
268.	Szczecin (Wzgórze zamkowe)	late 12th – 13th	zachodniopomorskie	loco
269.	Szczecin, 6 (E, W suburbium)	11th – mid 13th	zachodniopomorskie	loco
270.	Szczuka, 1	late 11th – mid 12th	kujawsko – pomorskie	Brodnica
271.	Szeligi, 1	6th – 7th	mazowieckie	Ożarów Mazowiecki
272.	Szeligi, 2a	6th – 7th	mazowieckie	Ożarów Mazowiecki
273.	Szeligi, 2b	6th – 7th	mazowieckie	Ożarów Mazowiecki
274.	Szestno, 3	10th/11th	warmińsko – mazurskie	Mrągowo
275.	Szurpiły	9th – 13th	podlaskie	Jeleniewo
276.	Świelubie, 1	7th – 9th/10th	zachodniopomorskie	Dygowo
277.	Tarnawa, 1	mid 9th – mid 10th	lubuskie	Rzepin
278.	Tądów Dolny, 2	late 11 – 13th	łódzkie	Warta
279.	Topolno	10th – 13th	kujawsko – pomorskie	Pruszcz
280.	Toruń – Zamek, 1	10th – 13th	warmińsko – mazurskie	loco
281.	Truszki Zalesie, 3 – 4	9th/10th – 11th/12th	podlaskie	Kolno
282.	Trzcianka	11th – 13th	podlaskie	Janów
283.	Trzemsze	13th/14th	wielkopolskie	Dobra
284.	Tum k. Łęczycy, 1	late 10th – mid 14th	łódzkie	Góra Św. Małgorzaty
285.	Tykocin, 1 – 2	12th – 13th	podlaskie	loco
286.	Tyłowo	9th	pomorskie	Krokowa
287.	Ujście, 1	late 10th – 15th	wielkopolskie	loco

No	Locality/site	Chronology	Province	Community
288.	Ujście, 5	10th – 14th	wielkopolskie	loco
289.	Warszawa – Wilanów, 13	11th/12th – 13th	mazowieckie	loco
290.	Węgry, 1	11th/12th	pomorskie	Sztum
291.	Wielowieś Ołobok, 23	10th – 13th	wielkopolskie	Sieroszewice
292.	Wierzchaczewo, 2	12th	wielkopolskie	Kaźmierz
293.	Więcbork	8th – early 12th	kujawsko – pomorskie	loco
294.	Wiślica	10th – 13th	świętokrzyskie	loco
295.	Włocławek, 1	9th – 12th	kujawsko – pomorskie	loco
296.	Wola Siennicka, 9	8th – 11th	lubelskie	Siennica Różana
297.	Wolin – miasto, 1/4 – 5	9th – 13th	zachodniopomorskie	Wolin
298.	Wolin – Przedmieście, 2	10th – 13th	zachodniopomorskie	Wolin
299.	Wolin – Srebrne Wzgórze, 5/I – II	11th – 13th	zachodniopomorskie	Wolin
300.	Wolin (Srebrne wzgórze)	9th – 13th	zachodniopomorskie	loco
301.	Wolin, 1	9th – 13th	zachodniopomorskie	loco
302.	Wolin, 10 (Osada południowa)	9th – 13th	zachodniopomorskie	loco
303.	Wolin, 2 (Przedmieście S)	10th – 13th	zachodniopomorskie	loco
304.	Wolin, 3	10th – mid 11th	zachodniopomorskie	loco
305.	Wolin, 4 (Ogrody)	9th – 13th	zachodniopomorskie	loco
306.	Wolin, 5	9th – 14th	zachodniopomorskie	loco
307.	Wrocław, 1 (Ostrów Tumski)	late 10th – 13th	dolnośląskie	loco
308.	Wrocław, Nowy Targ	12th – 14th	dolnośląskie	loco
309.	Wrocław, Ołbin	11th – 12th	dolnośląskie	loco
310.	Zawada, 1	9th – 11th	lubuskie	Zielona Góra
311.	Złotów, 3	8th – early 14th	wielkopolskie	loco
312.	Zwiniarz, 1	12th – 12th/13th	warmińsko – mazurskie	Grodziczno
313.	Żmijewo, 3 – 4	7th – 8th	kujawsko – pomorskie	Zbiczno
314.	Żółte, 1B	late10th – 10th/11th	zachodniopomorskie	Drawsko
315.	Żółte, 33	late 10th – 11th/12th	zachodniopomorskie	Drawsko
316.	Żółwin, 29	6th – 8th	lubuskie	Zwierzyn

No	Locality/site	Chronology	Province	Community
317.	Żukowice, 1	6th – 7th	dolnośląskie	loco
318.	Żukowice, 9	6th – 7th	dolnośląskie	loco
